# Identification of Genes Encoding Seminal Fluid Proteins in *Coccinella septempunctata* and Functional Analysis of the Gene Encoding Trypsin

**DOI:** 10.3390/insects17101023

**Published:** 2026-10-03

**Authors:** Ying Cheng, Yuhang Zhou, Changgeng Dai, Haiyan Ran, Cao Li

**Affiliations:** 1 Institute of Plant Protection, Guizhou Provincial Academy of Agricultural Sciences, Guiyang 550006, China; 2 Guizhou Branch of State Key Laboratory for Biology of Plant Diseases and Insect Pests, Guiyang 550006, China; 3 Guizhou Provincial Laboratory of Green Technology and Application Engineering of Plant Protection, Guiyang 550006, China; 4 Guizhou Provincial Pollution-Free Engineering Center of Plant Protection, Guiyang 550006, China

**Keywords:** ladybug, seminal fluid protein, transcriptome, proteome, trypsin

## Abstract

In this study, transcriptomic and proteomic analyses were used to identify genes encoding seminal fluid proteins in *Coccinella septempunctata* L. A total of 65 SFPs domains containing secretory signal peptides were identified, including trypsin, Kazal_2, His_Phos_2, Peptidase_M14, lipase, Kunitz_BPTI, Ribonuc_2-5A, and Gly-co_transf_90; an additional 22 signal peptide-containing domains were identified that lacked annotations in the Pfam database. Transcript levels of the 65 SFPs accounted for 87.58% of the total expression levels of 211 specifically expressed proteins in the male reproductive tract (MRT). RNA interference (RNAi) was used to analyze the function of the SFP trypsin. RNAi-mediated knockdown of the seminal fluid trypsin gene in male *C. septempunctata,* followed by mating with females, significantly reduced female fecundity and egg hatching rates. This study enriches our knowledge of insect SFPs and provides a theoretical basis for the artificial rearing of *C. septempunctata*.

## Simple Summary

Seminal fluid proteins (SFPs) function as key effectors in insects and induce changes in physiology and behavior after mating. The ladybird beetle, *Coccinella septempunctata* L. (Coleoptera: Coccinellidae), is a predatory enemy of aphids and plays a key role in biological pest control. Reports describing genes encoding SFPs in ladybirds and their regulatory effects on reproduction are limited. In this study, transcriptomic and proteomic approaches were used to identify SFP-encoding genes in *C. septempunctata*, and RNAi was utilized to analyze the effect of a gene encoding seminal fluid trypsin on reproductive function. The results of this study enrich our understanding of the regulatory mechanisms that operate in the reproductive function of male ladybirds and further contribute to enhancing the rearing of this important predator.

## 1. Introduction

Male insects transfer seminal fluids to females during mating, and these fluids contain sperm, seminal fluid proteins (SFPs) and small non-proteinaceous molecules. The SFPs produced by male accessory glands (MAG) function as the primary effectors and induce a series of behavioral and physiological changes [1]. For example, SFPs can function as chemical signals that regulate oocyte allocation in females, and these signals can result in increased egg production. Furthermore, the nutrients contained in ladybug seminal fluids can function as a supplemental energy source in females [2].

A prerequisite for determining the function of insect SFPs is to understand their composition. Various methods, including the use of suppressive subtractive hybridization libraries, microarrays, transcriptomics, and proteomics, have been used to identify SFPs in Coleoptera, Diptera, Hemiptera, and Hymenoptera, and the number of SFPs has varied from dozens to hundreds [3,4,5,6]. Since SFPs are secreted, screening for sequences encoding conserved secretion signals is often used to identify SFP-encoding genes in male insects [7]. Various approaches, including the injection of purified SFPs, ectopic expression experiments, and RNA interference (RNAi), have confirmed that SFPs promote sperm transfer and storage, increase female egg production, and enhance immunity [1,8]. For example, seminal fluid trypsins in *Drosophila melanogaster* and *Bombyx mori* participate in stimulating female egg production after mating and in sperm activation, respectively [9,10]. In *Aedes aegypti* males, mutations in the gene encoding leucine aminopeptidase 1 (*LAP1*) led to mitochondrial defects and abnormal autophagy in sperm, and this significantly reduced fertility and egg hatching rates in wild-type females mated with the defective males [11]. Furthermore, a SFP containing a trypsin inhibitor with a cysteine-rich domain induced changes in the transcriptome of *D. melanogaster* females [12].

The ladybird beetle, *Coccinella septempunctata* L. (Coleoptera: Coccinellidae), is a predacious enemy of aphids and plays a key role in biological pest control; consequently, methods that enhance reproductive efficiency result in larger ladybug populations and greater efficacy in controlling aphid pests. Studies on the regulation of reproductive function in *C. septempunctata* have primarily focused on females and have addressed vitellogenin synthesis, oogenesis, and oviposition [13,14,15]. In contrast, little is known about the systemic regulatory mechanisms that control male reproduction, including the functions of key genes and proteins. Insect SFPs are diverse, and research on the mechanistic basis of male reproduction is limited to a few species. Reports describing genes encoding SFPs in ladybirds and their regulatory effects on reproduction are limited. In this study, transcriptomics and proteomics were combined to select proteins that were present in male accessory glands and mated female tissues but absent in unmated female tissues and then filters SFPs by the presence of a signal peptide, and RNAi was utilized to analyze the effect of a gene encoding seminal fluid trypsin on reproductive function. The results of this study enrich our understanding of the regulatory mechanisms that operate in the reproductive function of male ladybirds and further contribute to enhancing the rearing of this important predator.

## 2. Materials and Methods

### 2.1. Insects

The *C. septempunctata* colony used in this study was collected from an experimental wheat field located at the Guizhou Academy of Agricultural Sciences. *C. septempunctata* was reared for 25 generations with *Aphis craccivora* serving as a food source. Rearing conditions were as follows: relative humidity 70% ± 5%; temperature 25 ± 1 °C, and photoperiod 14 L: 10 D.

### 2.2. Sample Collection

Female and male *C. septempunctata* adults were collected at 10 d post-eclosion and dissected in PBS buffer (137 mM NaCl, 2.68 mM KCl, 8.1 mM Na_2_HPO_4_, 1.47 mM KH_2_PO_4_, pH 7.4) and examined with a stereomicroscope. Whole male reproductive tracts (MRT) containing testes (TE), seminiferous ducts (SD), and male accessory glands (MAG), and whole female reproductive tracts (FRT) containing ovaries, bursa copulatrix, spermatheca, isolated MAGs, and female bursa copulatrix were dissected, washed in PBS, and stored at −80 °C. The samples used for transcriptome/proteome analysis contained 40 adult tissues, with 3 replicates for each sample.

### 2.3. Transcriptome Sequencing

#### 2.3.1. cDNA Library Construction and Transcriptome Sequencing

Total RNA was extracted from the MAGs, bursa copulatrix and spermatheca using Trizol (Promega, Beijing, China) reagent according to the manufacturer’s instructions. RNA purity and integrity were assessed using a NanoDrop spectrophotometer (Thermo Fisher Scientific, Waltham, MA, USA) and an Agilent 2100 Bioanalyzer (Agilent Technologies, Santa Clara, CA, USA), and RNA degradation was examined by gel electrophoresis in 1.5% agarose. cDNA libraries were constructed using the VAHTS Universal V10 RNA-seq Library Prep Kit (Vazyme, Nanjing, China). mRNA was enriched using oligo(dT) magnetic beads and randomly fragmented using divalent cations in NEB Fragmentation Buffer. Using the fragmented mRNA as a template and random oligonucleotides as primers, first-strand cDNA was synthesized with M-MLV reverse transcriptase. The RNA strand was degraded with RNaseH, and second-strand cDNA was synthesized using dNTPs as substrates in a DNA polymerase I. The ends of purified double-stranded cDNA were repaired, polyA-tailed, and ligated to sequencing adapters. cDNA fragments of approximately 150 bp were selected using AMPure XP beads, amplified by PCR, and the resulting PCR products were purified again using AMPure XP beads to obtain the final library. Quantification and quality control were performed using an Agilent 2100 Bioanalyzer and qRT-PCR. Qualified libraries were sequenced on an Illumina NovaSeq X Plus platform.

#### 2.3.2. Sequence Annotation and Gene Expression Analysis

Raw reads obtained from the Illumina NovaSeq X Plus platform were filtered for quality using fastp v0.23.2 [16]. The reference genome and gene model annotation files were downloaded from a genome website (https://ftp.ncbi.nlm.nih.gov/genomes/all/GCF/907/165/205/GCF_907165205.1_icCocSept1.1/GCF_907165205.1_icCocSept1.1_genomic.fna.gz, accessed on 24 February 2025). An index of the reference genome was built using HISAT2 v2.2.1 [17], and paired-end clean reads were aligned to the reference genomeusing HISAT2 v2.2.1. Annotation was performed against NR [18], UniProt [19], KEGG (kobas v3.0) [20], GO (https://pubmed.ncbi.nlm.nih.gov/10802651, accessed on 26 February 2025), and other databases. The program feature Counts v2.0.1 [21] was used to calculate the number of reads mapped to each gene. After the quantification of gene expression was completed, statistical analysis was performed to screen for genes with significant differences in expression levels among samples under different states. The fragments per kb per million fragments (FPKM) [22] was used to calculate unigene expression [23]. Differential expression analysis between two comparison groups was performed using DESeq2 v1.16.1 [24]. Genes with |log2(FoldChange)| > 1.0 & adjusted *p*-value < 0.05 were considered differentially expressed.

### 2.4. Proteome Sequencing

Data-independent acquisition (DIA-NN, v1.8.1) [25] was employed for quantitative proteomics sequence analysis. Total protein concentration of tissue samples was determined using a bicinchoninic acid (BCA) kit (Shanghai Biyuntian Biotechnology Co., Ltd., Shanghai, China). DIA analyses of samples were performed using the Vanquish Neo UHPLC System (Thermo Fisher Scientific, Lenexa, KS, USA) for chromatographic separation, and the separated samples were analyzed using an Orbitrap Astral Mass Spectrometer (Thermo Fisher Scientific, Lenexa, KS, USA). The search method was Libraryfree, and the database was the uniprot-proteome.fasta database. The setting ‘match between runs’ was used to generate a spectral library from the DIA data, and the library was then used to re-analyze the DIA data and quantify proteins. False discovery rates (FDR) for protein levels were filtered at 1%, and the data were then used in bioinformatics analyses.

### 2.5. Identification of Seminal Fluid Proteins in C. septempunctata

SignalP 5.0 (www.dtu.dk/services/SignalP/, accessed on 13 May 2025) [26] was used to predict secretory signal peptides in proteins. Proteins that were detected in mated-FRT and MAG samples but not in unmated-FRT samples were identified, and those that contained secretory signals were designated as seminal fluid proteins.

### 2.6. Real-Time Quantitative PCR Analysis

*C. septempunctata* adults were collected at 10 d after eclosion and dissected to obtain MRT, FRT, MAG and SD, and each sample contained tissues from 20 adults. Total RNA was extracted from each sample using the Eastep^®^ Super Total RNA Extraction Kit (Promega, Beijing, China), and cDNA was synthesized using the iScript cDNA Synthesis Kit (Bio-Rad, Hercules, CA, USA). Primers were synthesized by Sangon Biotech (Shanghai) Co., Ltd. (Shanghai, China) (Table 1). Primers for 20 different target genes were designed based on the transcriptome database, and the internal control primers (Actin-F/Actin-R) were designed and synthesized as described previously [27]. The PCR reaction system (20 μL) consisted of the following: 2 μL cDNA template, 2 μL each of forward and reverse primers, 10 μL Sso Advanced Universal SYBR Green Supermix (Bio-Rad, USA), and 4 μL ddH_2_O. PCR was conducted as follows: 95 °C for 3 min; 40 cycles of 95 °C for 5 s and annealing at the respective primer-specific temperature for 30 s. Melting curves were generated to verify primer specificity. Each biological replicate contained three.

### 2.7. RNAi of the Trypsin Gene

#### 2.7.1. dsRNA Synthesis

The *C. septempunctata* genomes database (https://ftp.ncbi.nlm.nih.gov/genomes/all/GCF/907/165/205/GCF_907165205.1_icCocSept1.1/GCF_907165205.1_icCocSept1.1_genomic.gff.gz, accessed on 13 May 2025) was used to obtain the coding sequence for trypsin (accession no. LOC123308654). Specific primers for trypsin-dsRNA (Tr-dsRNA) were designed within the coding region of the trypsin gene using ClustalX and Primer 5.0, and T7 promoter sequences were added to both ends of each primer (Table 1). Specific primers for the gene encoding green fluorescent protein (*GFP*) are shown in Table 1. dsRNA synthesis was performed as described in Cheng et al. [29].

#### 2.7.2. dsRNA Injection

Newly emerged, 1-d-old ladybird males were injected with 1 μL of Tr-dsRNA (1000 ng/μL) using a microinjection system (Eppendorf Transferman 4R, Eppendorf, Hamburg, Germany). Injection methods followed those described previously [29], and males injected with GFP-dsRNA served as controls. Each treatment consisted of 50 males with three replicates.

#### 2.7.3. Effects of RNAi

Following injection with dsRNAs, male ladybirds were reared on aphids (*A. craccivora*). At 5 and 10 d after injection, male adults were dissected to collect MRTs for RNA extraction, cDNA synthesis, and RT-qPCR. Each sample contained three biological replicates, and each replicate consisted of MRTs from 10 males. On days 5 and 10 after dsRNA injection, testes were dissected from male adults in PBS buffer, and testes were observed using a stereomicroscope. Images were captured using ImageView software (x64, 4.11.18709.20210403) and testes lengths and widths and accessory gland lengths were determined. Thirty males were dissected for each treatment.

#### 2.7.4. Fecundity Statistics

Injected males were paired with virgin females of the same age. There were 5 pairs in each treatment, and treatments were replicated five times. A total of 25 pairs were analyzed, and egg production and hatching rates were recorded daily for 20 d after injection.

### 2.8. Data Analysis

One-way analysis of variance (ANOVA) was used to analyze data in three samples, and differences between two samples were compared for significance using the *t*-test (comparison between two sets of data) or multiple tests (comparison among the three sets of data) in DPS v. 19.05 [30].

## 3. Results

### 3.1. Transcriptome Analysis

As shown in Table 2, a total of 248,275,863 raw reads were obtained. After filtering with fastp (0.23.2), 247,974,517 clean reads were obtained (File 1). These high-quality sequences were aligned to the reference genome using HISAT2 v2.2.1, and this resulted in 173,474,889 mapped reads and 154,871,523 reads with unique alignment positions. There may be differences between the published reference genome samples of *C. septempunctata* and the individual samples sent for sequencing in this experiment. Quantitative analysis of gene expression levels for each sample yielded a total of 16,412 genes (File 2). Among these, 11,356 genes were expressed in unmated bursa copulatrix and spermatheca (unmated-BCS) samples, 11,182 in mated bursa copulatrix and spermatheca (mated-BCS) samples, and 12,090 in MAG samples.

### 3.2. Identification of Seminal Fluid Proteins

Transcriptomic and proteomic analyses identified 13,769 transcripts and 5760 proteins, and 5754 genes were represented in both the transcriptome and proteome (Figure 1A, File 3). We used a quadrant plot to show the differences in unmented-BCS_vs_mated-BCS (Figure 1B), identifying 144 genes (blue dots in the top right and bottom right quadrants) that showed significant differences at the transcriptomic level, and 621 proteins (blue dots in the top left and top right quadrants) that showed significant differences at the proteomic level. Among them, 30 genes and protein expression trends were consistently upregulated (top right quadrant). Volcano plots can visually display the distribution of differentially expressed genes for each comparison combination (Figure 2). Among those, 144 were upregulated and 165 were downregulated in the mated-BCS group compared with the unmated-BCS group (Figure 2A), 1500 were upregulated and 1761 were downregulated in the unmated-BCS group compared with the MAG group (Figure 2B), and 1454 were upregulated and 1713 were downregulated in the mated-BCS group compared with the MAG group (Figure 2C). SignalP v5.0 predicted 2057 secretory signal peptides among the detected proteins (File 4). To search and analyze the proteomic database of MAG, mated-BCS, and unmated-BCS samples, the relative quantitative mean value of a protein within an experimental group was set at 314.2005 as the low-peak protein (this protein was determined to be undetected). A total of 211 proteins were present in both MAG and mated-BCS samples but were absent from unmated-BCS samples. The 211 proteins were successfully transferred to the female ladybug through mating. Among these 211 proteins, 65 of them contain secretory protein signal peptides that were likely part of functional SFPs (File 5). These included five trypsins, four Kazal_2 proteins, three His_Phos_2 proteins, three peptidase_M14 proteins, two lipases, two Kunitz_BPTI proteins, one Ribonuc_2-5A protein, one Glyco_transf_90 protein, and 22 signal peptide-containing proteins without annotations in the Pfam database. Male accessory glands are the primary site where SFPs are produced, and the SFPs were highly abundant in MAGs (Figure 3). The 65 SFPs (File 5) were highly protein abundances in the MAGs of unmated 10-d-old *C. septempunctata* males and accounted for 87.58% of the total abundances of the 211 proteins detected in MAGs (*t* = 0.27, *df* = 4, *p* = 0.8058) (Figure 3). In mated-BCS, the protein abundances of these 65 SFPs accounted for 83.11% of the total protein abundances of the 211 proteins (*t* = 1.50, *df* = 4, *p* = 0.2088).

### 3.3. RT-qPCR Analysis of SFPs

RT-qPCR was used to analyze the expression patterns of genes encoding 20 putative SFPs (File 5) in MRT, FRT, and dissected MRT tissue that contained seminiferous ducts, testes and MAGs. Among these 20 genes (Figure 4, File 6), 16 showed specific or enhanced expression in MRT. Expression levels of three trypsin genes (*LOC123308654*, *LOC123314887*, and *LOC123319134*) were significantly higher in MRT than in FRT (*t* = 8.38, *df* = 4, *p* = 0.0011; *t* = 10.15, *df* = 4, *p* = 0.0005; *t* = 5.61, *df* = 4, *p* = 0.0050). The lipase-encoding gene *LOC123308194* was expressed at levels nearly 7000-fold higher in MRT than in FRT (*t* = 12.67, *df* = 4, *p* = 0.0002), which indicated tissue-specific expression. Three Kazal_2 domain protein-encoding genes (*LOC123307234*, *LOC123317366*, and *LOC123317428*) showed significantly higher expression in MRT than in FRT (*t* = 7.73, *df* = 4, *p* = 0.0015; *t* = 8.37, *df* = 4, *p* = 0.0011; *t* = 6.20, *df* = 4, *p* = 0.0034); among these, *LOC123317428* expression was nearly 10,000-fold higher in MRT compared with FRT, indicating tissue-specific expression in MRT. However, four genes encoding Peptidase_S8 (*LOC123310785*), Glyco_transf_90 (*LOC123311794*), poly(A)-specific ribonuclease PARN isoform X2 (*LOC123316586*), and Pkinase (*LOC123317163*) (*t* = 3.73, *df* = 4, *p* = 0.0202; *t* = 7.72, *df* = 4, *p* = 0.0015; *t* = 12.16, *df* = 4, *p* = 0.0003; *t* = 15.34, *df* = 4, *p* = 0.0001) showed higher expression in FRT than in MRT. When MRT tissues were dissected and analyzed for gene expression, the 20 genes showed either MAG-specific or biased expression levels (Figure 5, File 6). Only Carb_anhydrase (*LOC123309449*) (F(2,4) = 63.85, *p* = 0.0009) and Peptidase_M14 (*LOC123315231*) (F(2,4) = 4.49, *p* = 0.0450) had higher expression levels in the TE as compared with MAG and SD. Expression of the gene encoding Ldl_recept_a (*LOC123322097*) (F(2,4) = 8.52, *p* = 0.0362) was higher in SD than in MAG and TE. In summary, the results of RT-qPCR were consistent with gene expression patterns obtained from the transcriptome.

### 3.4. Functional Analysis of the SFPs Trypsin

#### 3.4.1. Effect of RNAi on the Expression of the Trypsin Gene in *C. septempunctata*

*Trypsin* expression decreased significantly after males were injected with Tr-dsRNA (Figure 6, File 7). At 5 d after injection, *Trypsin* expression was decreased by 35-fold in MRT treated with Tr-dsRNA compared with the control group injected with GFP-dsRNA (*t* = 13.85, *df* = 4, *p* = 0.0002). On day 10 after injection with Tr-dsRNA, *Trypsin* expression decreased by 36-fold compared with the group injected with GFP-dsRNA (*t* =8.64, *df* = 4, *p* = 0.0010). These results indicate that injection with Tr-dsRNA silenced the expression of *Trypsin* in MRT.

#### 3.4.2. Effect of RNAi on Testes Development in *C. septempunctata*

When testes were examined 5 d after Tr-dsRNA injection (Figure 7A, File 7), testes length (Tl) (*t* = 5.02, *df* = 4, *p* = 0.0074), width (Tw) (*t* = 7.15, *df* = 4, *p* = 0.0020), and accessory gland length (Agl) (*t* = 10.95, *df* = 4, *p* = 0.0004) were significantly lower than the GFP-dsRNA injected group. At 10 d after Tr-dsRNA injection (Figure 7B, File 7), testes length (*t* = 1.24, *df* = 4, *p* = 0.2817), testes width (*t* = 0.42, *df* = 4, *p* = 0.6977) and accessory gland length (*t* = 2.03, *df* = 4, *p* = 0.1118) were comparable to those in the GFP-dsRNA group, and there were no significant differences among the two groups.

#### 3.4.3. Effect of RNAi on Fecundity in *C. septempunctata*

When evaluated over a 20-d period, the mean number of eggs laid per female after mating with Tr-dsRNA-injected males was 97, whereas egg production for the GFP-dsRNA control group was 228 (Figure 8A, File 7). Egg production in the Tr-dsRNA group was significantly reduced (*t* = 3.0892, *df* = 8, *p* = 0.0149) relative to the GFP-dsRNA control (Figure 7A). The egg hatching rate in the Tr-dsRNA group was 35.40% and was significantly lower than the GFP-dsRNA group, which had a hatching rate of 96.80% (*t* = 4.70, *df* = 8, *p* = 0.0015) (Figure 8B, File 7).

## 4. Discussion

The presence or absence of a signal peptide is a classic method for determining whether a protein is secreted [7]. In *Nilaparvata lugens*, 94 proteins were identified as SFPs and 84 contained signal peptides [7]. In *A. aegypti* and *Aedes albopictus*, 93 and 198 SFPs were identified, with 29 and 57 of them containing signal peptides, respectively [31]. In this study, transcriptomics and proteomics were used to identify SFPs in *C. septempunctata*, and 211 proteins were present simultaneously in MAGs and mated-BCS. This demonstrates that the 211 protein genes are indeed transferred to the female during mating. Among the 211 protein-encoding genes, 65 contained secretion signal peptides, and these included genes encoding lipase, trypsin, serine protease, glycosyltransferase, and ribonuclease. The remaining 147 predicted proteins had no obvious signal peptides due to a missing N-terminal sequence in the protein database; thus, it remains unclear whether this group includes unidentified secreted proteins.

SFPs represent a major contribution to reproduction, and several studies have demonstrated the role of male-specific SFPs in sexual selection and reproduction in animals [1,32,33,34]. In *Tribolium castaneum*, knockdown of the gene encoding seminal angiotensin-converting enzyme 9 in males resulted in decreased egg production in females after mating [3]. In *Spodoptera frugiperda*, knockout of the serine protease encoded by *SPSL1* led to male sterility but did not affect sperm production or migration in the male reproductive system [35]. In *Ophraella communa*, the hexokinase-encoding *HK2* gene was specifically expressed in male testes and regulated the expression of an Apolipophorin-like (*Ap-like*) gene. *Ap-like* activated transcription of the gene encoding triacylglycerol lipase, which caused changes in triglyceride levels in the reproductive system and fat body, thereby regulating female reproductive capacity [33]. In crickets, seminal trypsin has a role in stimulating female egg-laying during mating [36], whereas seminal trypsin functions in sperm activation in *B. mori* and *Manduca sexta* [10,37]. In *N. lugens*, the expression of 16 genes encoding seminal trypsin was simultaneously inhibited by RNAi, but it did not lead to a decrease in the egg production of the female after mating [7]. In this study, RNAi knockdown of seminal trypsin (LOC123308654) in *C. septempunctata* males significantly inhibited fecundity in females, further confirming that the expression of seminal trypsin has a role in regulating female reproductive behavior. We also found that silencing the trypsin gene in males affected the development of the testes and accessory glands, with gradual recovery of development by 10 days after injection. This led to a transient developmental deficit at the time of mating; this might also be the reason for the reduced egg production and decreased hatching rate. Meanwhile, only the gene with the highest expression level among the seminal trypsin was selected for RNAi in this study, which the phenotype could still involve paralogs or a transient developmental effect. Insect mating induces transcriptional changes in numerous genes in female insects, including genes involved in immune responses, chemosensation, and metabolism [38]. In *A. aegypti*, SFPs were shown to induce transcriptomic changes in the female reproductive tract [39]. We plan to use transcriptomics combined with RNAi to explore how seminal trypsin protease regulates the reproductive pathway in *C. septempunctata* females.

The functions of these SFPs in *C. septempunctata* will be elucidated in future studies. With the rapid development of technologies such as proteomics, metabolomics, and gene editing, studies focusing on the functions of SFPs in agricultural pests and natural enemies will help resolve the complex functions of SFPs in insect reproduction. Ultimately, this strategy will provide new perspectives and theoretical foundations for studying reproductive biology in insects and may lead to new approaches for pest management.

In this study, 43 seminal fluid proteins genes as homology-based PFAM/NR predictions were identified in *C. septempunctata*. The functions of the seminal fluid proteins His_Phos_2, Kazal_2, Peptidase_M14, lipase, Kunitz_BPTI, Ribonuc_2-5A, and Glyco_transf_90 have not been well-studied. Among these, the genes encoding lipase and Kazal_2 were specifically expressed in the testes of *C. septempunctata* and were also highly expressed in MAGs. Furthermore, our study also revealed 22 newly discovered genes without prior functional annotation. The seminal fluid trypsin gene regulates the reproductive function of female *C. septempunctata*.

## Figures and Tables

**Figure 1:**
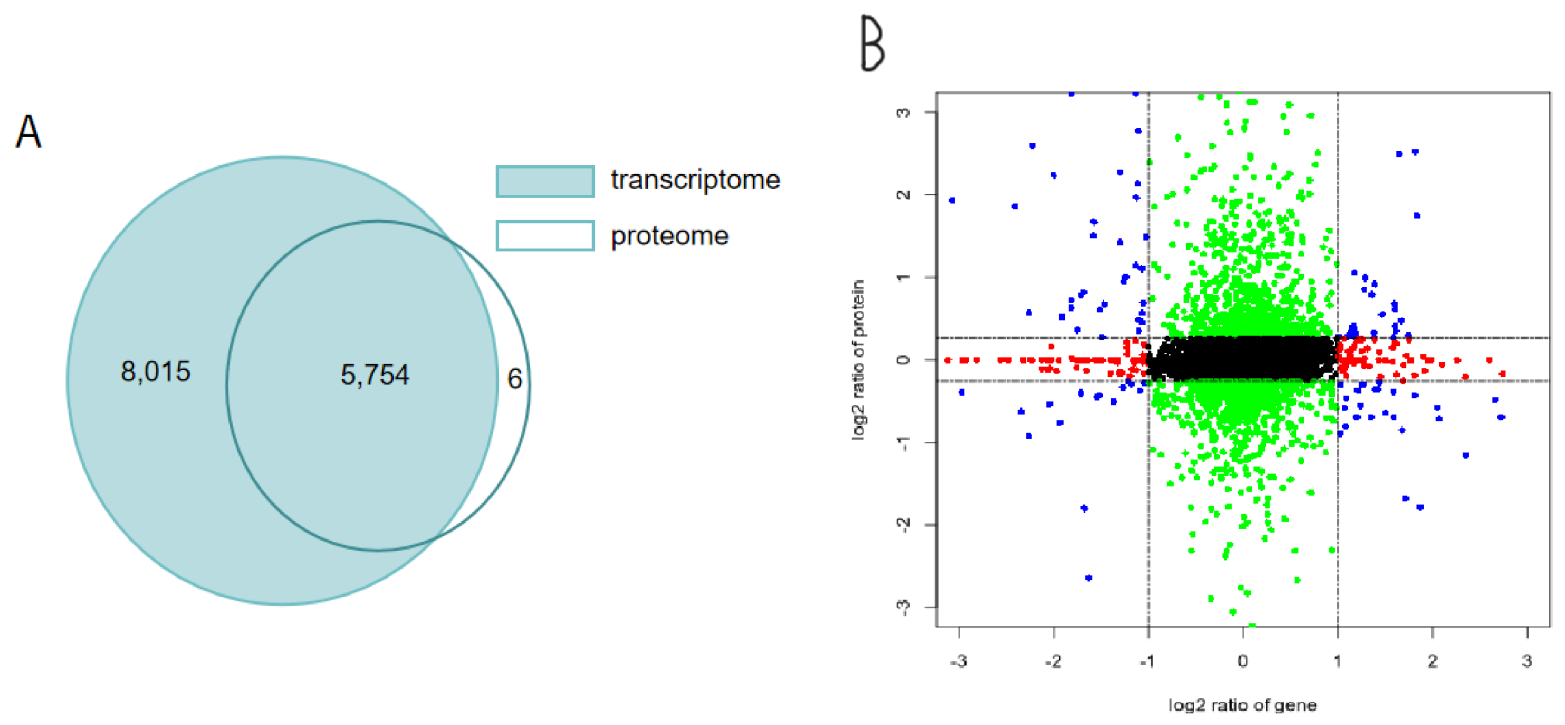
Comparative Venn (**A**) and Quadrant (**B**) diagram of the *Coccinella septempunctata* transcriptome and proteome.

**Figure 2:**
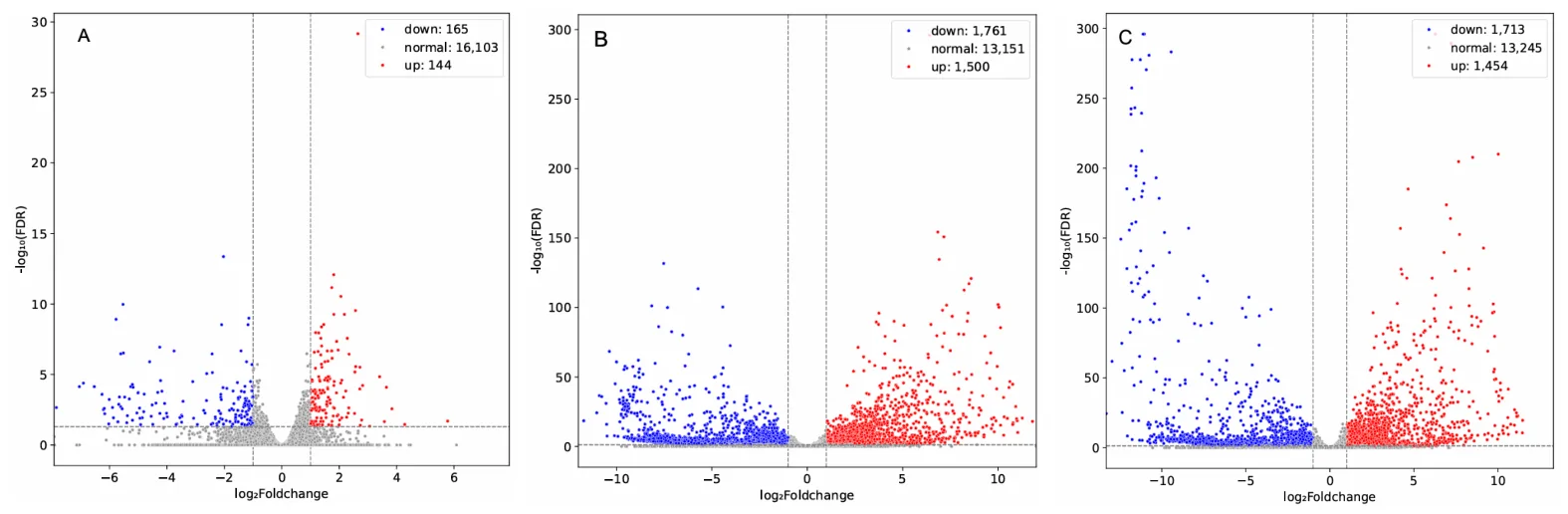
The differential expression analysis of RNA-seq data in different reproductive organs of *Coccinella septempunctata*. (**A**) unmated-BCS_vs_mated-BCS, (**B**) MAG_vs_unmated-BCS, (**C**) MAG_vs_mated-BCS.

**Figure 3:**
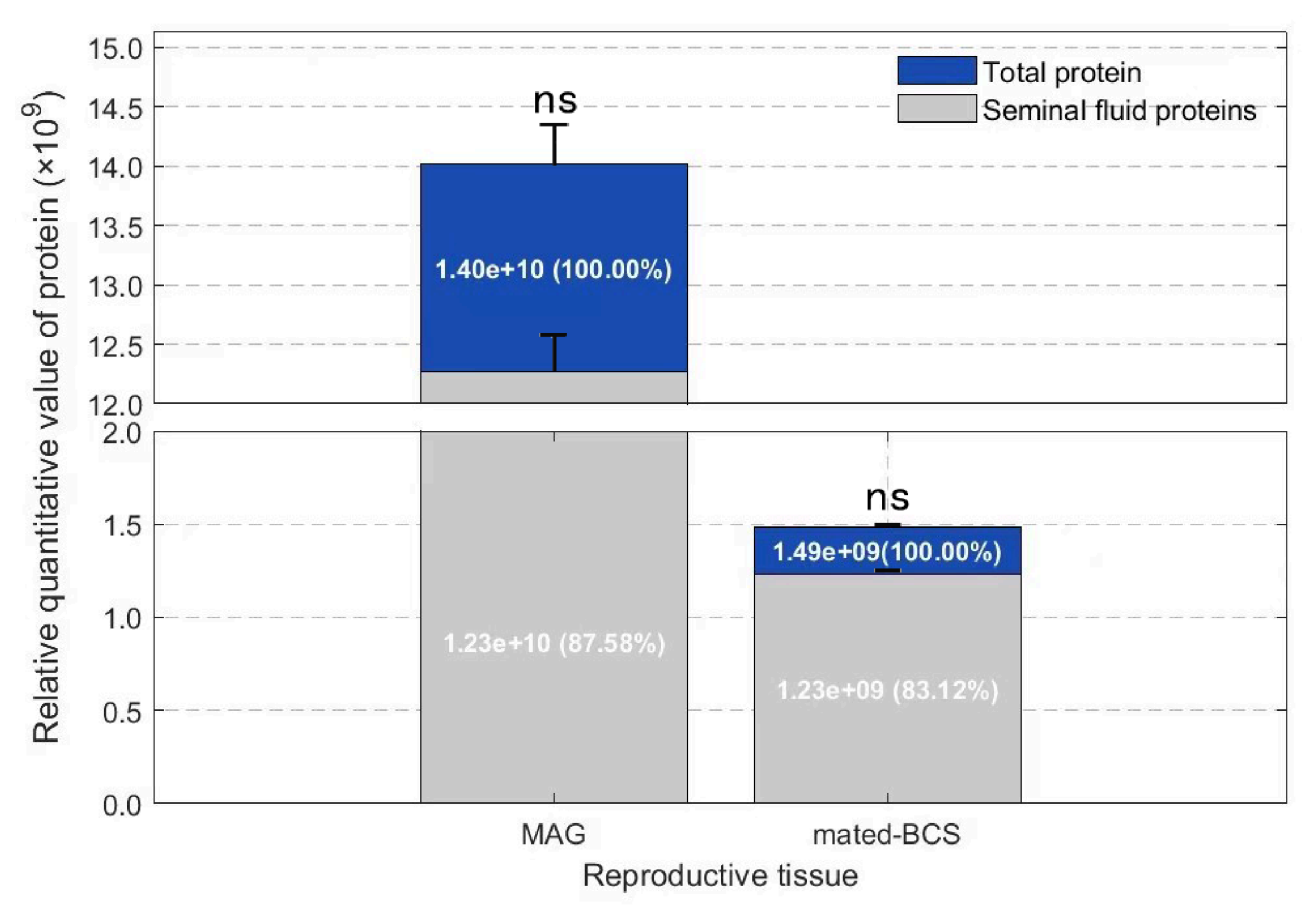
Relative quantitative value of proteins in male accessory glands (MAG) and mated bursa copulatrix and spermatheca (mated-BCS) of *Coccinella septempunctata*. ns, the difference is not significant. Data are means ± SD.

**Figure 4:**
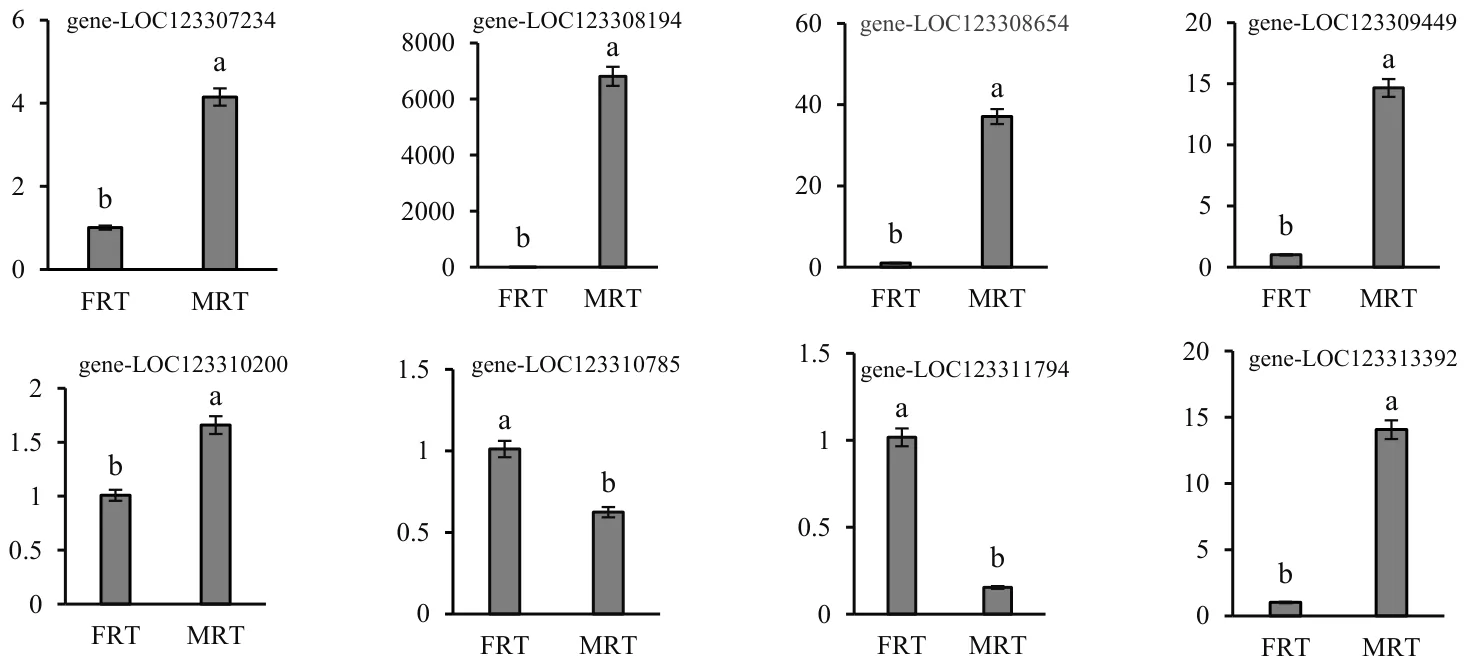

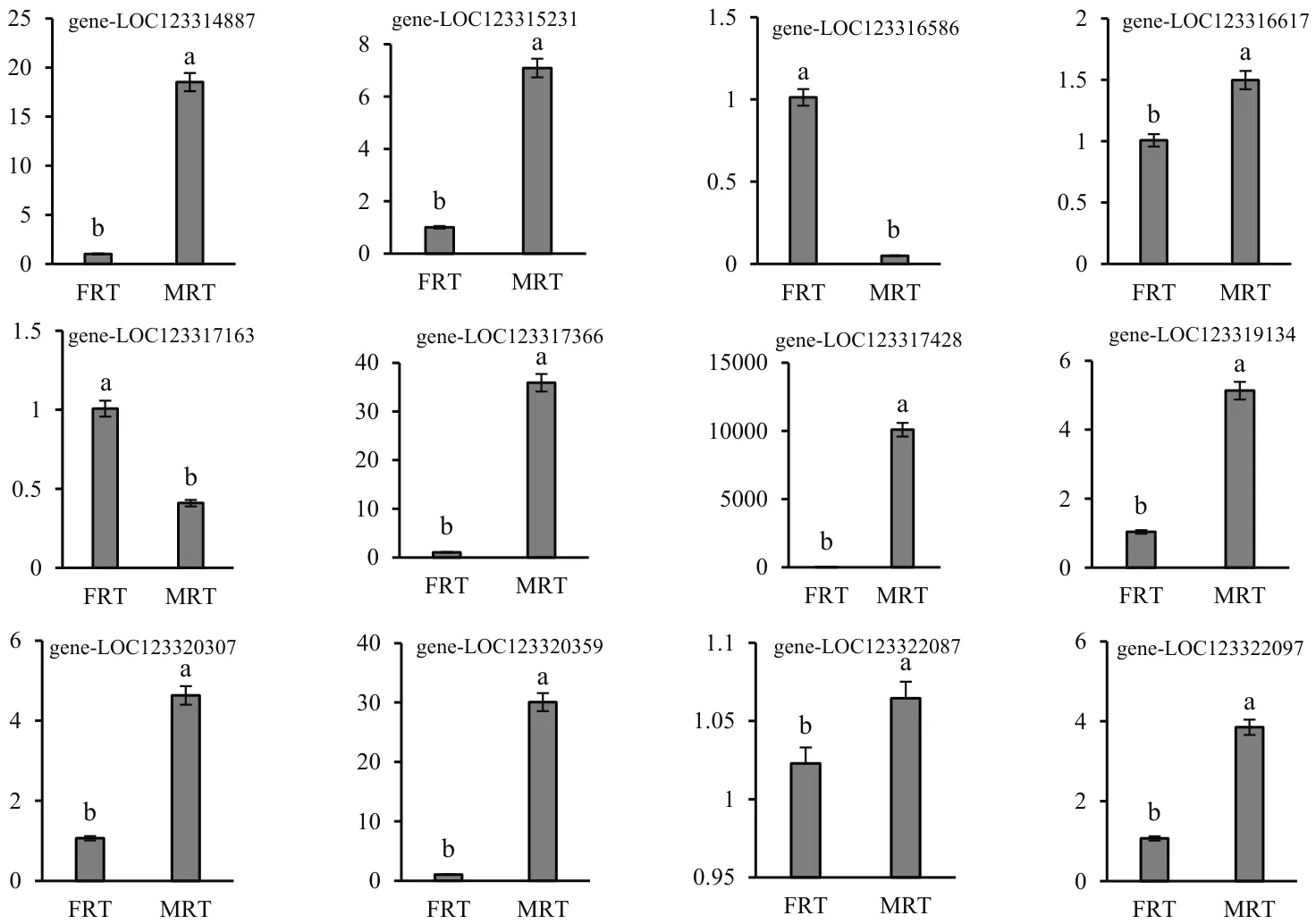
Expression of genes encoding putative seminal proteins in the female (FRT) and male (MRT) reproductive tracts of *Coccinella septempunctata*. Ordinate column: the relative expression level of genes. Data represent means ± SD. Columns labeled with different letters indicate significance at *p* < 0.05 using the *t*-test.

**Figure 5:**
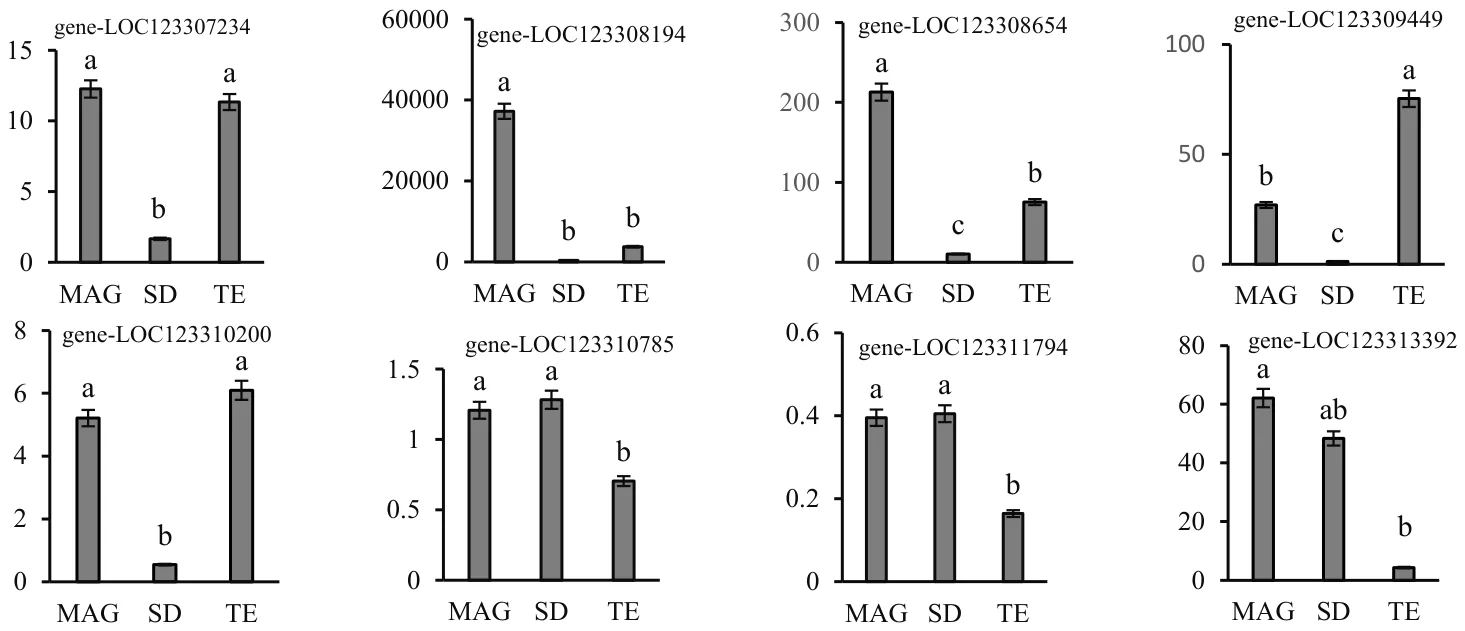

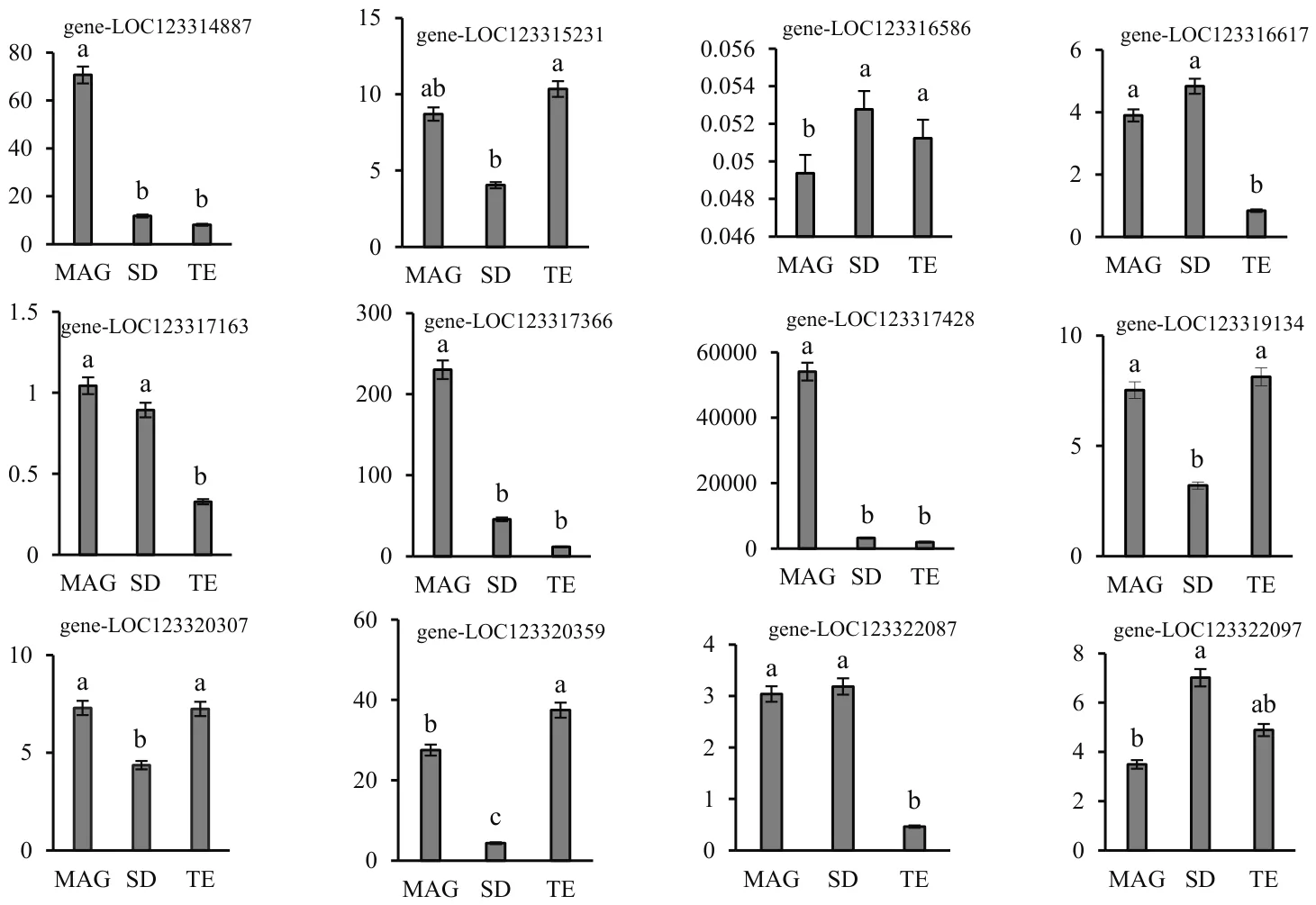
Tissue-specific expression of genes encoding seminal fluid proteins in *Coccinella septempunctata.* Ordinate column: the relative expression level of genes. Abbreviations: MAG, male accessory glands; SD, seminiferous ducts; and TE, testes. Data represent means ± SD. Columns labeled with different letters indicate significance at *p* < 0.05 using the LSD test.

**Figure 6:**
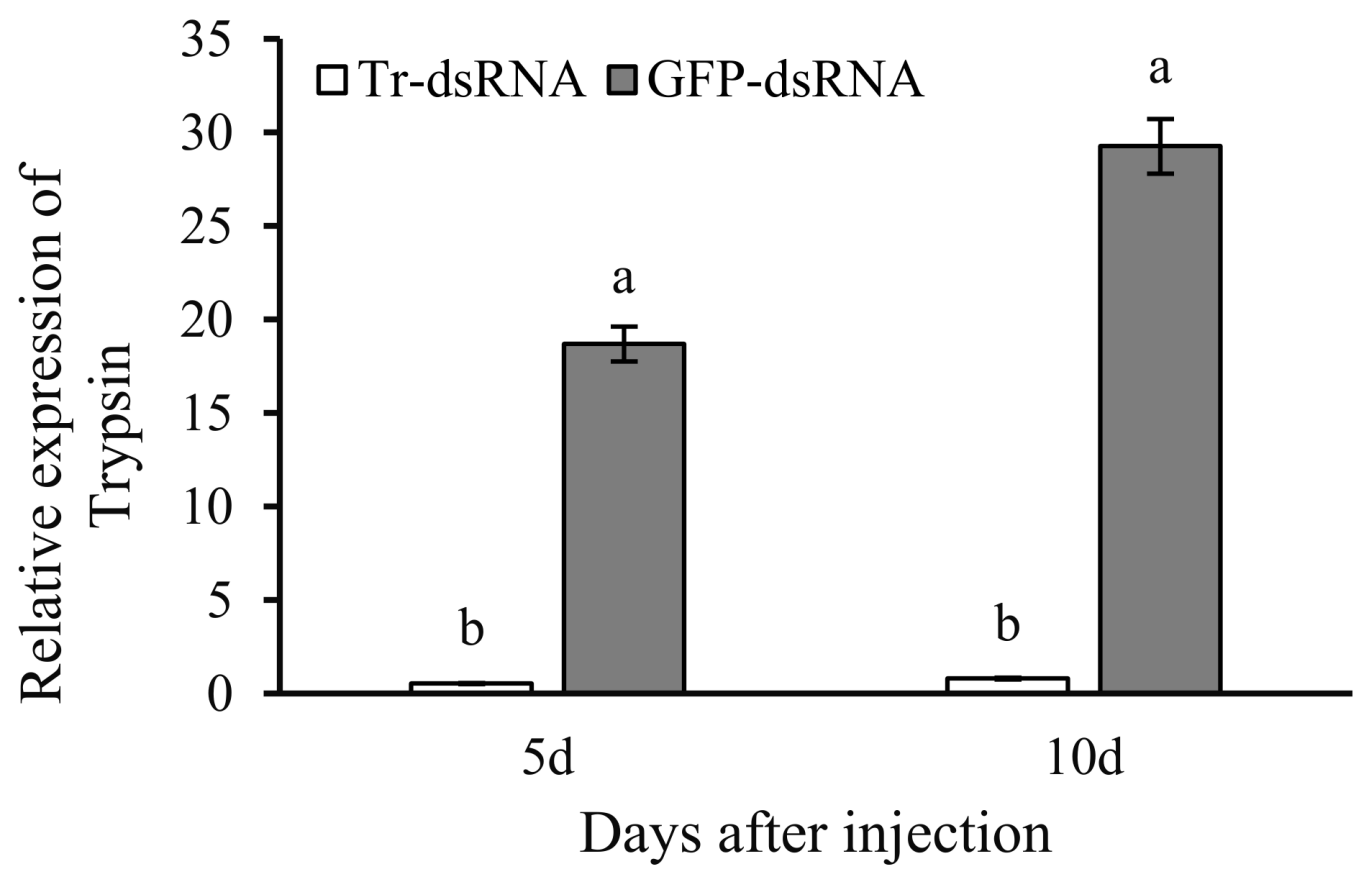
Relative expression levels of *Trypsin* in *Coccinella septempunctata* after injection with Tr-dsRNA and GFP-dsRNA. Each sample contained three biological replicates, and each replicate consisted of MRTs from 10 males. Data represent means ± SD, and columns labeled with different letters indicate significance at *p* < 0.05 using the *t*-test.

**Figure 7:**
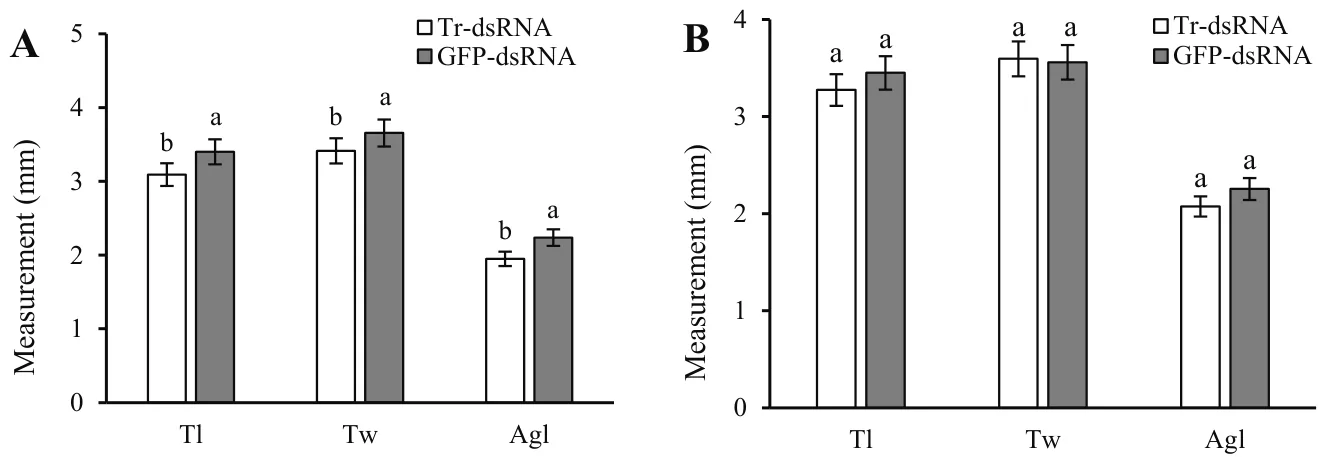
Testes measurements in *Coccinella septempunctata* after injection with Tr-dsRNA and GFP-dsRNA. (**A**) Testes measurements at 5 d after microinjection and (**B**) testes measurements at 10 d. Abbreviations: Tr-dsRNA, trypsin dsRNA; GFP-dsRNA, green fluorescent protein dsRNA; Tl, testes lengths; Tw, testes widths; and Agl, accessory gland length. Data represent means ± SD. Columns labeled with different letters indicate significance at *p* < 0.05 using the *t*-test.

**Figure 8:**
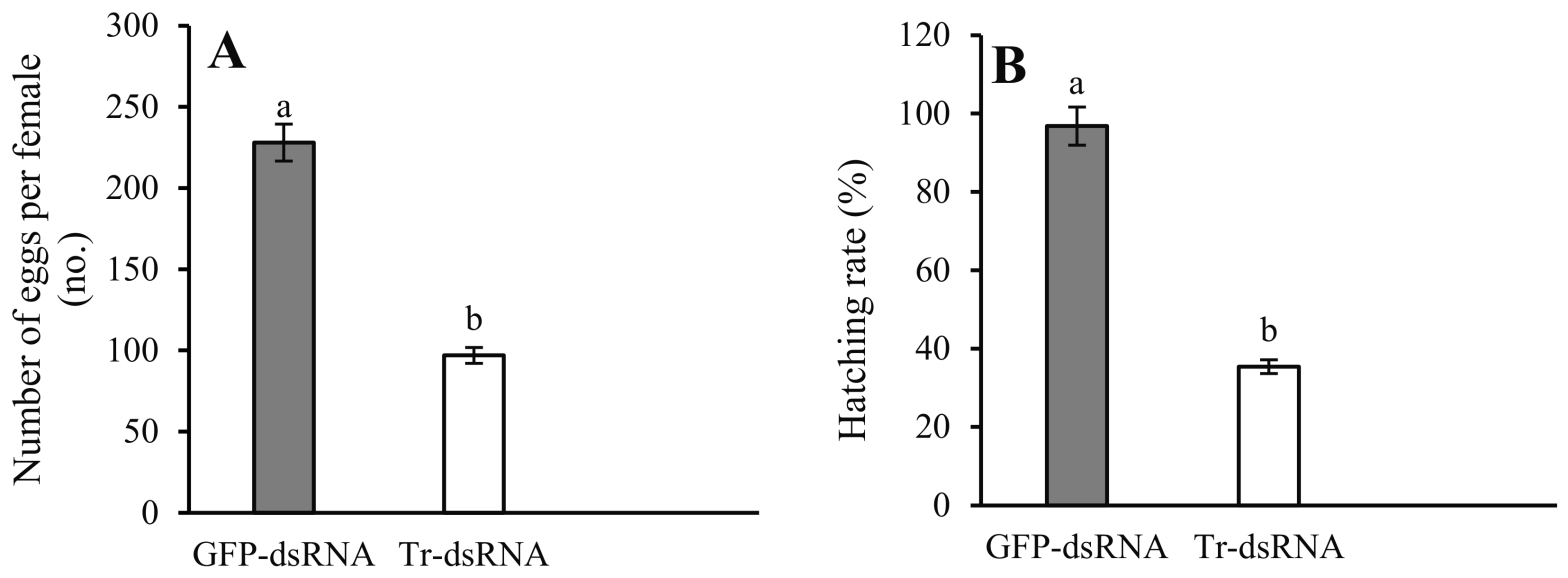
Egg production (**A**) and hatching rates (**B**) in *Coccinella septempunctata* after Tr-dsRNA injection. Abbreviations: Tr-dsRNA, trypsin-dsRNA; GFP-dsRNA, green fluorescent protein dsRNA. Data represent means ± SD. Columns labeled with different letters indicate significance at *p* < 0.05 using the *t*-test.

**Table 1:** Primers used in this study (5′–3′).

ID	Primer Sequence (5′ to 3′)	ID	Primer Sequence (5′ to 3′)
LOC123307234	CCCAACCTACTTCGACCCAGT	LOC123316586	GCAAAGAAGCTCAAATGACGC
	GCAGGAACCGAGGTGTTTCAT		TGGTCACGAGAAGGATTAGGG
LOC123308194	GTCGTCCATACCAGCTCTTTCT	LOC123316617	CATCAAGCGATACGACGGGAA
	CCGTTGATGGTTACACACGAAG		GTTCCACCACAGCCACACTC
LOC123308654	TGGGATATTCACATCGGTCCAT	LOC123317163	TGGACAACACAAATCAGGGCT
	TCGGTTTAATGCTCAGTTCTCC		CTGGAATTGGTACGTCAGGGT
LOC123309449	CCCAACCAAACTACTGCCAAG	LOC123317366	AGGACTGTGTGGAGAGATGTC
	TGTCCATACTACCACGTCGTT		CGTGGTATGCAACTTGGACAC
LOC123310200	CCATCCTCCTTCCGAATGCTTC	LOC123317428	GCACAGCACCAGGTTTAACAA
	CAGCGGTTTAACATGATTGCCC		GTTCAGTGTTCCTCGTGTCCT
LOC123310785	CTCCTTTCACTTGTTTTGCCGC	LOC123319134	ACCTTTTAGTGTTGTCGCAGGA
	ACAGACAAGCCACTATCAACGT		AGTTCACGCTCCAGTTCGATAA
LOC123311794	TGCATTCGTTTGGGCATTGATT	LOC123320307	GTGAAATGCAATCCGTCCTCG
	TCCAATATGTCAGTCTGCGTGT		CAACCTGGGCAGTCTTCAGAT
LOC123313392	CTGCTGCGACAATTCAACATGA	LOC123320359	TGGACTCTACCCACCTGTTGA
	TTTCCTCTGCTTGTTCCAGTGA		TGGACGCAGGAAATTGTTTGC
LOC123314887	GCTCAGGAGATAGTGGTGGC	LOC123322087	AGAACACACAGGCATCAACGTT
	AGCTTTCTTCACCGATGCGT		TCCCGGCAAAGTGAAATTGTGT
LOC123315231	ATGAGCAGGATCGTATGTGGT	LOC123322097	GCCTTCTCCCTTCACACGTTC
	GTCCCAGTTAATAGCCGCATC		GTTTGCACCGTTTCGTTAGCC
Actin	GATTCGCCATCCAGGACATCTC		
	TCCTTGCTCAGCTTGTTGTAGTC		
Tr-dsRNA	taatacgactcactatagggCGTTTGGGAGAAACAACGAT †		
	taatacgactcactatagggTTTCAGACCTTCTGGTCGCT †		
GFP-dsRNA	taatacgactcactatagggGCCAACACTTGTCACTACTT †		
	taatacgactcactatagggGGAGTATTTTGTTGATAATGGTCTG †		

† Nucleotides in lowercase represent the T7 promoter region. Technical replicates and the relative expression levels of target genes were calculated using the 2^−∆∆Ct^ method [28].

**Table 2:** Transcriptome analysis of the *Coccinella septempunctata* reproductive system.

Summary	Number
Total number of reads	248,275,863
Total number of clean reads	247,974,517
Total number of maps	173,474,889
Total number of unique maps	154,871,523
Total number of genes	16,412
Unmated bursa copulatrix and spermatheca expression	11,356
Mated bursa copulatrix and spermatheca expression	11,182
Male accessory gland expression	12,090

## Data Availability

The data that support the findings of this study are openly available in Zenodo at https://doi.org/10.5281/zenodo.22254760. The raw sequence data reported in this paper have been deposited in the Genome Sequence Archive (Genomics, Proteomics & Bioinformatics 2025) at the National Genomics Data Center (Nucleic Acids Res 2025), China National Center for Bioinformation/Beijing Institute of Genomics, Chinese Academy of Sciences (GSA: CRA050414) that are publicly accessible at https://ngdc.cncb.ac.cn/gsa, accessed on 13 May 2025.
